# Long-term agro-management strategies shape soil bacterial community structure in dryland wheat systems

**DOI:** 10.1038/s41598-023-41216-z

**Published:** 2023-08-25

**Authors:** Shikha Singh, Surendra Singh, Scott B. Lukas, Stephen Machado, Amin Nouri, Francisco Calderon, Elizabeth R. Rieke, Shannon B. Cappellazzi

**Affiliations:** 1 Hermiston Agricultural Research and Extension Center, Oregon State University, 2121 S 1St, Hermiston, OR 97838 USA; 2 Columbia Basin Agricultural Research Center, Oregon State University, 48037 Tubbs Ranch Rd., Adams, OR 97810 USA; 3 Soil Health Institute, 2803 Slater Rd, Morrisville, NC 27560 USA; 4 GO Seed, 4455 60th Ave NE, Salem, OR 97305 USA

**Keywords:** Agroecology, Microbial ecology

## Abstract

Soil microbes play a crucial role in soil organic matter decomposition and nutrient cycling and are influenced by management practices. Therefore, quantifying the impacts of various agricultural management practices on soil microbiomes and their activity is crucial for making informed management decisions. This study aimed to assess the impact of various management systems on soil bacterial abundance and diversity, soil enzyme activities and carbon mineralization potential in wheat-based systems. To accomplish this, soil samples from 0 to 15 cm depth were collected from ongoing long-term field trials in eastern Oregon region under wheat (*Triticum aestivum* L.)-fallow (WF), WF with different tillage (WT), wheat-pea (*Pisum sativum* L.) (WP), WF under different crop residue management (CR) and natural undisturbed/unmanaged grassland pasture (GP). These trials consisted of an array of treatments like tillage intensities, nitrogen rates, organic amendments, and seasonal residue burning. This study was a part of the Soil Health Institute’s North American Project to Evaluate Soil Health measurements (NAPESHM). Bacterial community structure was determined using amplicon sequencing of the V4 region of 16SrRNA genes and followed the protocols of the Earth Microbiome Project. In addition, extracellular enzyme activities, and carbon mineralization potential (1d-CO_2_) were measured. Among different trials, 1d-CO_2_ in WT, WP, and CR studies averaged 53%, 51% and 87% lower than GP systems, respectively. Enzyme activities were significantly greater in GP compared to the other managements and followed similar trend as respiration. We observed higher evenness in GP and higher richness in spring residue burning treatment of CR study. Our results indicated that species evenness is perhaps a better indicator of soil health in comparison to other indices in dryland wheat systems.

## Introduction

Soil is a dynamic and complex living system encompassing a diverse suite of physical, chemical, and biological attributes. These attributes play a direct role in agricultural productivity and overall ecosystem functioning^1, 2^. Soil microbial communities play a crucial role in performing soil functions such as: decomposition of organic matter, nutrient mineralization and cycling^3, 4^, promoting biogenic stability of soil structure which improves water infiltration and root growth^5^, and additional services such as acting as food resources for other species in the soil environment^6^. Recently, there has been growing interest in assessing soil health measurements, especially soil biological components to understand soil’s capacity to function. Since microbes play a vital role in ecosystem functioning, it is important to assess their activity and community structure in relation to soil health and potential functions. In soil ecosystems, soil microbes are decomposers that metabolize, uptake and turnover soil organic carbon (SOC) by secreting extracellular enzymes to hydrolyze organic polymers into monomers and plant available nutrients. Therefore, soil enzyme activities are used as an indicator of soil’s capacity to cycle nutrients. They can be informative because of their quick responses to stress and management practices, easy measurement, and integrative nature^7^. Other properties such as microbial respiration (carbon mineralization potential), and diversity are also considered important indicators of soil health^8^. Carbon mineralization potential is generally thought to be indicative of overall potential microbial activity while diversity indices are useful to further understand the status of the microbial communities in soil. For instance, species richness shows the number of different species while evenness refers to the relative abundance of species within an environment^9^.

Growing demand for food has resulted in the conversion of unmanaged and pristine systems into arable lands and the need for higher yields has led to use of intensive management practices such as tillage, residue removal, residue burning, and extensive inorganic fertilizer applications. These intensive practices and overuse lead to deterioration of soil health, soil structure, and loss of fertility^10^. For instance, intensive tillage and residue removal/burning may lead to soil erosion^11^, and loss of biodiversity^12^. These management practices are key drivers of microbial communities like bacteria and strongly impact biodiversity and ecosystem functioning^13–15^. Therefore, using these indicators in determining soil health can substantially improve soil health assessment frameworks^16^.

Understanding the soil microbial dynamics is crucial as well as challenging, mostly due to high diversity and the complex nature of soils. Agricultural practices alter the soil environment and can significantly affect the microbial community. However, the effects of these management practices on potential microbial activity (carbon mineralization potential) vary depending on several other factors such as soil type, crop rotation, climate, and management history. For instance, conservation practices such as no-till (NT) systems have shown greater microbial activity^17–20^ compared to conventional tillage systems. Similarly, some studies have reported higher enzyme activities under no-till systems, but the trends are variable and inconclusive^21, 22^. Residue management practices have also been reported to alter bacterial community structure^23^. Navarro-Noya, et al.^24^ reported that residue retention led to increased Bacteroidetes and Betaproteobacteria and decreased Cyanobacteria and Gemmatimonadetes. Conservation agricultural practices, in previous studies, have been shown to favor oligotrophic bacteria (Acidobacteria, Planctomycetes, and Verrucomicrobia) while conventional practices favorcopiotrophic bacteria (Actinobacteria)^25^. This is likely due, in part, to mixing of top soil layers by conventional tillage practices thereby making more organic substrates and oxygen available for microbes^26^. Likewise, burning crop residue is also reported to alter bacterial community structure^27^.

Despite knowing that agricultural management practices strongly affect the soil bacterial community and overall microbial activity, little information is available on how long-term implementation of these practices (tillage intensities, nitrogen rates, residue management and organic inputs) affect the microbial activity and bacterial diversity in the dryland wheat (*Triticum aestivum* L.) systems of inland Pacific Northwest region. Therefore, this study was conducted as a compliment to the Soil Health Institute’s North American Project to Evaluate Soil Health Measurements (NAPESHM)^28^ to assess the long-term impacts of various management systems on soil bacterial abundance and diversity, soil enzyme activities and carbon mineralization potential in wheat-based systems. Results were also compared with a long-term undisturbed grassland established in 1930. We hypothesized that the bacterial activity and bacterial diversity will be significantly affected by the management practices and that unmanaged grasslands will exhibit the highest bacterial diversity. More specifically, more destructive tillage practices like moldboard plow and chisel till will exhibit lower bacterial diversity than no till and no till with cover crops treatments.

## Materials and methods

### Study sites

This study leveraged a long-term undisturbed grassland pasture (GP) and three long-term ongoing wheat (*Triticum aestivum* L.) systems field studies, viz. wheat-tillage (WT), wheat-pea (*Pisum sativum* L.) (WP) and crop residue management (CR) (Table 1). All the three long-term experiments are established at the Oregon State University’s Columbia Basin Agricultural Research Center (CBARC). All the studies comply with the institutional, national, and international guidelines and legislation. The GP, WP, and CR studies are located at CBARC Pendleton, OR and WT study is located at the CBARC Moro, OR. The GP has been under undisturbed mixed species native perennial grass dominated by Bluebunch wheatgrass (*Agropyron spicatum* Pursh) and Idaho fescue (*Festuca idahoensis* Pursh). The soil at these experimental sites is classified as loess-derived well-drained Walla Walla silt loam (coarse-silty, mixed, superactive, mesic Typic Haploxeroll—US; Kastanozems—FAO). The Pendleton region experiences mean annual precipitation of 437 mm and mean maximum and minimum annual temperatures of 17.4 °C and 3.06 °C, respectively. The Moro region receives 280 mm average annual precipitation and mean maximum and minimum annual temperatures of 15.6 °C and 3.39 °C, respectively.

**Table 1:** Description of study sites, cropping systems and treatments.

Field Studies	Location	Treatments and abbreviations
Grassland pasture (GP) 1931-present	Oregon State University’s Columbia Basin Agricultural Research Center (CBARC) at Pendleton, OR. (45°42′N, 118°36′W, elevation 438 m asl)	• GP: The grassland pasture has been under undisturbed/unmanaged mixed species native perennial grass dominated by
		◦ Bluebunch wheatgrass (*Agropyron spicatum Pursh*)
		◦ Idaho fescue (*Festuca idahoensis Pursh*)
Wheat-Pea (WP) 1963- current	CBARC at Pendleton, OR. (45°42′N, 118°36′W, elevation 438 m asl)	• CTWP: Chisel tilling to 20 cm depth (twice) in fall + sweeping to 5 cm depth + roto-tilling twice to 10 cm depth
		• PTWP: Moldboard plowing to 20 cm depth in fall + disc harrowing to 10 cm depth (twice) + roller disc harrowing
		• NTWP: No tillage + rotary mowing of wheat stubble + sweeping to 5 cm depth
Crop Residue Management (CR) 1931—current	CBARC at Pendleton, OR. (45°42′N, 118°36′W, elevation 438 m asl)	• NB90: No residue burning + 90 kg N ha^−1^ (UAN) + moldboard plowing to 20 cm depth + harrowing to 10 cm depth + rod-till weeding (3 times)
		• NBFM: No residue burning + Farmyard Manure (FYM) at rate of 11.2 Mg ha^–1^ yr^–1^ (47.5% DM, 0.85 Mg C ha^–1^, and 70 kg N ha^–1^ yr^−1^) + moldboard plowing to 20 cm depth + harrowing 10 cm + rod-till weeding (3 times)
		• SB90: Spring burning of crop residue + 90 kg N ha^−1^ (UAN) + moldboard plowing to 20 cm depth + harrowing to 10 cm depth + rod-till weeding (3 times)
		• NBPV: No residue burning + Pea Vine at a rate of 1.12 Mg ha^–1^ yr^–1^ (Dry matter 87.8%, 0.41 Mg C ha^–1^ yr^–1^, and 18.5 kg N ha^–1^ yr^–1^) + moldboard plowing to
		• 20 cm depth + harrowing 10 cm + rod-till weeding (3 times)
Wheat-tillage (WT) 2003—present	CBARC at Moro, OR. 45°29′N and 120°43′W, elevation 575 m asl)	• NTWC: No tillage + drill seeding of wheat (240 seeds m^−2^) + winter pea cover crop
		• NTWF: No tillage + drill seeding of wheat (240 seeds m^−2^) + fallowing for 14 months
		• TTWF: Chisel tilling to 15 cm depth + sweeping to 10 cm depth + rod-till weeding to 10 cm depth (3 times) + drill seeding of wheat (240 seeds m^−2^) + fallowing for 14 months

#### Wheat-tillage (WT) study

This study was initiated in 2003 under 2-yr winter wheat-fallow (WF) system at the CBARC in Moro, OR (45°29′N120°43′W, elevation 575 m a.s.l.). Winter wheat was drill seeded at the rate of 240 seeds m^−2^ in September every year and harvested in the end of July followed by a fallow phase. For weed management, Glyphosate was applied at label rates ranging from 0.84 to 1.26 kg ha^−1^ acid equivalent. Treatments included Chisel (trash) till (TTWF), NT (NTWF), and NT with purple vetch (*Vicia benghalensis* L.) cover crop (NTWC) (Table 1). The treatments were arranged in a randomized complete block design with three replicates and details about the treatments are shown in Table 1. Nitrogen (N) and Sulfur (S) were applied during the wheat phase based on soil testing that ranged from 22 to 45 kg ha^−1^ for N and 4 to 13 kg ha^−1^ for S. Fertilizer was banded 2.5 cm below seed during planting. Further details on this field study can also be found in Machado, et al.^29^.

#### Wheat-pea (WP) study

This study was initiated in 1963 under wheat-pea rotation system. Treatments included chisel tillage (CTWP), moldboard plow (PTWP), and NT (NTWP) (Table 1) and the treatments were arranged in a split plot with four replicates. Each treatment plot was 27 m × 7.3 m with three replicates. All tillage operations were done in fall after harvest of the wheat crop every other year. Winter wheat was planted using a double disk drill in October and harvested in late July. Pea was sown in March–April and harvested in mid-July. All wheat plots received N at the rate of 90 kg ha^−1^ (ammonium nitrate and urea) and pea received 22 kg N ha^−1^ (ammonium phosphate sulfate) every wheat year. Weed control was achieved by applying Glyphosate at the rate of 314–628 g ha^−1^ acid equivalent. More detailed description about this study can be found in Shiwakoti, et al.^30^.

#### Crop residue (CR) management study

The CR study was established in 1931 under a winter wheat-fallow system. Treatments randomized block design with four replicates and are listed in Table 1. All treatments plots were 40.2 m × 11.6 m with four replicates. These plots were drill seeded at the rate of 90 kg ha^−1^ before 2002 and 92 kg ha^−1^ after 2002. Weed management was done by applying 2, 4-dichlorophenoxyacetic acid thrice at the rate of 0.88-L ha^−1^. Moldboard plowing to a 20 cm depth was completed after organic amendment application and residue burning. Further details about this study can be found in Shiwakoti, et al.^31^.

### Soil sampling and analysis

Soil samples were collected from all experimental sites in April 2019 from 0–15 cm depth, using a sharpshooter and soil knife to collect multiple subsamples homogenized to create a composite sample. The composite samples were immediately air-dried and sieved through an 8 mm sieve and processed accordingly for different soil analyses. A subsample of the fresh soil was shipped with ice packs in coolers within five days to the laboratories for enzyme and DNA extractions. More details on the sampling protocol for the NAPESHM project are reported in Norris, et al.^28^. Soil organic carbon (SOC) and total nitrogen (TN) were measured using dry combustion method^32^, soil available phosphorus (P) and potassium (K) were extracted using Mehlich III extraction method^33^, and soil pH was measured using 1:2 soil water suspension^34^. Similarly, soil electrical conductivity (EC) was measured using 1:2 soil water suspension^35^, and soil texture was determined by pipette and sieve method^36^. Carbon mineralization potential (mg CO_2_–C kg^−1^) was measured following the 24-h incubation of moist soil using an infrared gas analyzer (1d-CO_2_)^37^. Briefly, 40 g of dry soil sample is wetted to 50% of pore volume and then incubated in an airtight jar for 24 h. Following this, gas samples are collected and analyzed using an infrared gas analyzer. Extracellular enzyme activities including β-glucosidase and phosphatase were determined by assay incubation of fresh soil samples followed by colorimetric measurement^38, 39^. Bacterial community structure was determined using 16S rRNA amplicon sequencing of the V4 region. The entire process including DNA extraction, primer selection, library preparation and sequencing was conducted following the Earth Microbiome Project protocols^40, 41^. Briefly, DNA extraction was done using the DNeasy PowerSoil Pro Kit® (QIAGEN) following the manufacturer’s suggested protocol with slight modifications. Polymerase Chain Reaction (PCR) amplification of V4 region was done using the 515F and 806R primers^42^ modified with adapters on the 5ʹ end for the Illumina MiSeq platform. The PCR reaction mixture included 13 µL of PCR-grade water, 10 µL 2X PCR master mix, 0.5 µL of 10 µM of both forward and reverse primers, and 1 µL of template DNA. The PCR conditions were the following: initial denaturation at 94 °C for 3 min, followed by 35 cycles each at 94 °C for 45 s (denaturation), 50 °C for 60 s (annealing), and 72 °C for 90 s (elongation), followed by 72 °C for 10 min (final elongation). Results from PCR were confirmed by running each sample on a 1% agarose gel. Library preparation and sequencing was performed at Oregon State University’s Center for Qualitative Life Sciences. The sequenced data was then processed using R software^43^. Divisive Amplicon Denoising Algorithm 2 (DADA2), a parametric model was used to infer true biological sequences from reads and was run as an R script in R v.3.6 using dada2 package v.1.7. The sequences were quality filtered using the “filter and trim” function and error rates were determined from a set of few samples reads and also estimated separately for each sequencing run. The sequences were then deduplicated, denoised, merged, chimeras were removed, and Amplicon Sequence Variants (ASVs) were inferred at > 99% similarity. A total of 11,435 ASVs were obtained. The sequences were then taxonomically annotated using SILVA version 132 reference alignment to obtain the best species classification. The sequences were submitted to National Center for Biotechnology Information Sequence Read Archive with accession number PRJNA762046.

### Statistical analyses

The effects of various management practices on carbon mineralization potential and extracellular enzyme activities were analyzed using PROC GLIMMIX in SAS software v9.4 (SAS Institute Inc., 2002). The mean separation was done using Tukey’s HSD test and the mean differences were considered significant if *p* ≤ 0.05. To understand how the bacterial community varied among treatments, permutational multivariate analysis of variance (PERMANOVA) was used in R^43^. To identify the microbial classes that were significantly enriched in each treatment, we conducted the indicator species analysis in R. The *indval* score for each ASV is the product of their relative abundance and relative frequency within each treatment. Only the ASVs with a *p*-value ≤ 0.05 were considered significant for all treatments. The diversity indices including richness, evenness, inverse Simpson, and Shannon’s index were also determined using functions from the “vegan” package in R^43^. The relationship between soil bacterial phyla and soil chemical properties was determined through the redundancy analysis (RDA) performed using the “vegan” package in R. The Pearson correlations between soil properties and bacterial phyla were also calculated using PROC CORR in SAS software v9.4 to assess the linear dependency between two independent variables.

## Results

### Effects of various agromanagements on soil chemical characteristics

Among all the field studies, SOC (22.3 g kg^−1^) under unmanaged GP were the greatest and averaged 135%, 40%, and 96% higher than WT, WP, and CR studies, respectively (Table 2). Similarly, TN in GP was 2.5, 1.6, and 2.2-fold higher than WT, WP, and CR studies, respectively. Within WT and WP studies, both SOC and TN were similar among the treatments while within CR study, these were significantly higher in SB90 than the other treatments. Available P was significantly higher in SB90 (63.4 mg kg^−1^) than in all other treatments across all studies (Table 2). Comparing within experiments, no significant differences in treatments were observed in available P in WT and WP studies while within CR study, SB90 averaged > 100% higher available P than NBFM, NBPV, and NB90 treatments. Similarly, available K was highest in SB90 followed by GP while the lowest available K was observed in all treatments under WT study and treatments NB90, NBFM, and NBPV from CR study. Within studies, treatments of WT study had similar available K levels while in WP study, PTWP (668.8 mg kg^−1^) showed the lowest available K. The CR study trends in available K under different treatments were similar to available P. The pH values across all studies ranged from 4.6 to 6.6 (Table 2). No significant differences in EC were observed within WT study while in WP study, EC was significantly higher in NTWP than other treatments. In CR study, EC showed the following trend: SB90 > NMFM > NB90 = NBPV. No significant differences in clay content were found among treatments across all studies (Table 2).

**Table 2:** Soil characteristics under various long-term experiments.

		SOC	TN	Available P	Available K	pH	EC	Clay content
		(g/kg)		(mg/kg)			(mmho/cm)	(%)
Unmanaged	GP	22.3a^†^	2.0a	12.66b	795.5b	6.10b	0.160ab	11.60
WT study	TTWF	9.5d	0.8d	7.26b	507.2e	4.90fg	0.140bc	11.00
	NTWF	9.2d	0.8d	7.60b	519.7e	4.70fg	0.160ab	11.40
	NTWC	9.7d	0.8d	9.65b	549.8e	5.00efg	0.110bcde	11.50
WP study	CTWP	15.9bcAB	1.2c	3.30b	742.3bcA	4.90fgAB	0.110cdeB	15.10
	PTWP	14.7cB	1.1c	3.50b	668.8cdB	5.00efA	0.098deB	15.70
	NTWP	17.3bA	1.3b	3.74b	749.7bcA	4.66gB	0.190aA	13.90
CR study	NB90	9.6dB	0.7dC	2.50bB	521.5eB	5.80cB	0.034gC	12.30
	NBFM	10.6dB	0.9dB	1.90bB	581.3deB	5.30deC	0.080efB	11.96
	SB90	14.6cA	1.2bcA	63.40aA	1169.5aA	6.60aA	0.130bcdA	12.20
	NBPV	10.6dB	0.8dBC	1.50bB	564.9eB	5.50cdBC	0.040fgC	12.30

^†^Means followed by different lowercase letters within a column are statistically different across all field studies at *p* ≤ 0.05. Different uppercase letters within a column indicate significantly different means within each study at *p* ≤ 0.05. No uppercase letters mean no statistical significance.

### Carbon mineralization potential

**Figure 1:**
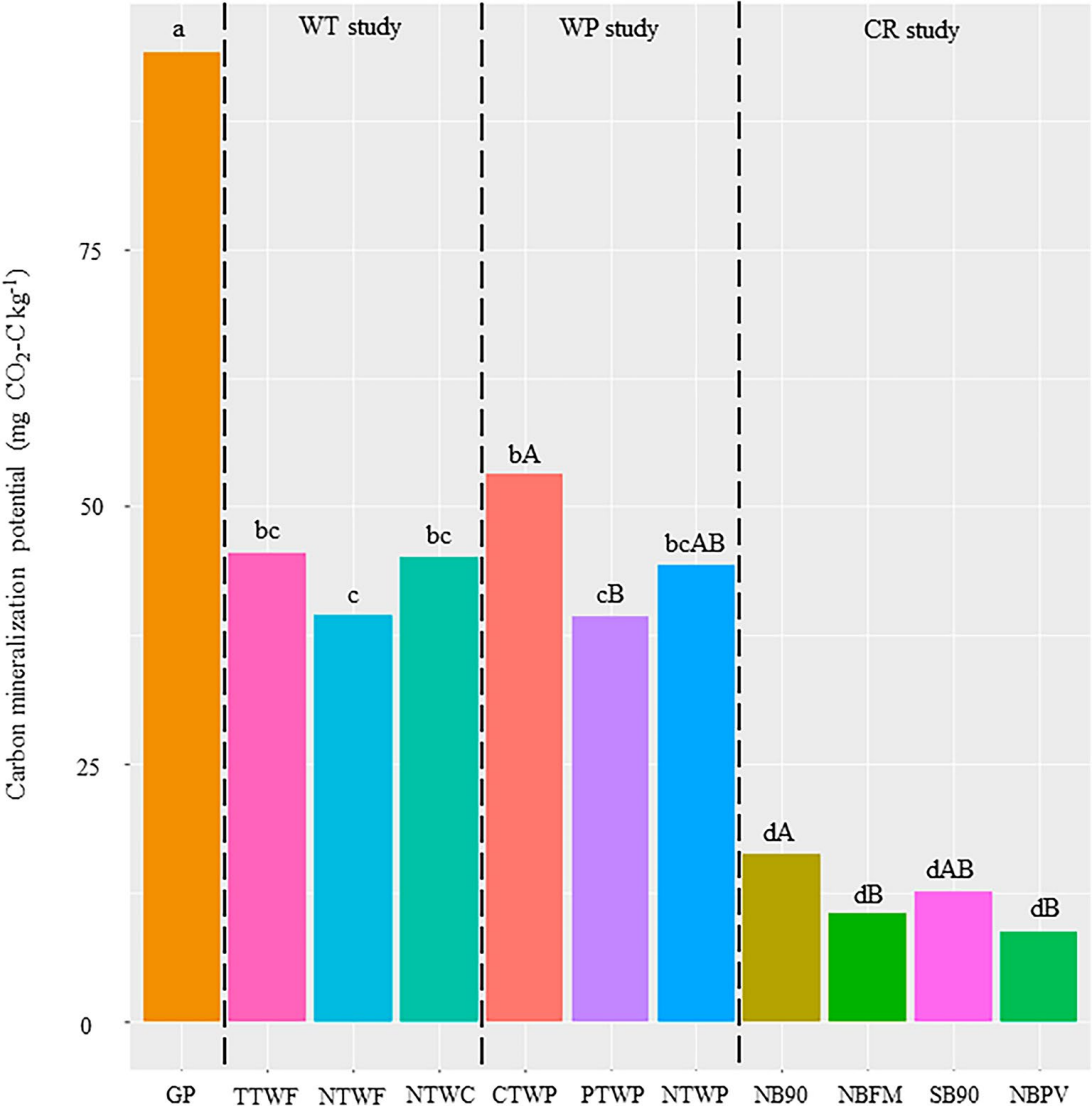
Carbon mineralization potential in various long-term experiments. Different lowercase letters represent significantly different means across all field studies at *p* ≤ 0.05. Different uppercase letters indicate significantly different means within each study at *p* ≤ 0.05. No uppercase letters mean no statistical significance.

**Figure 2:**
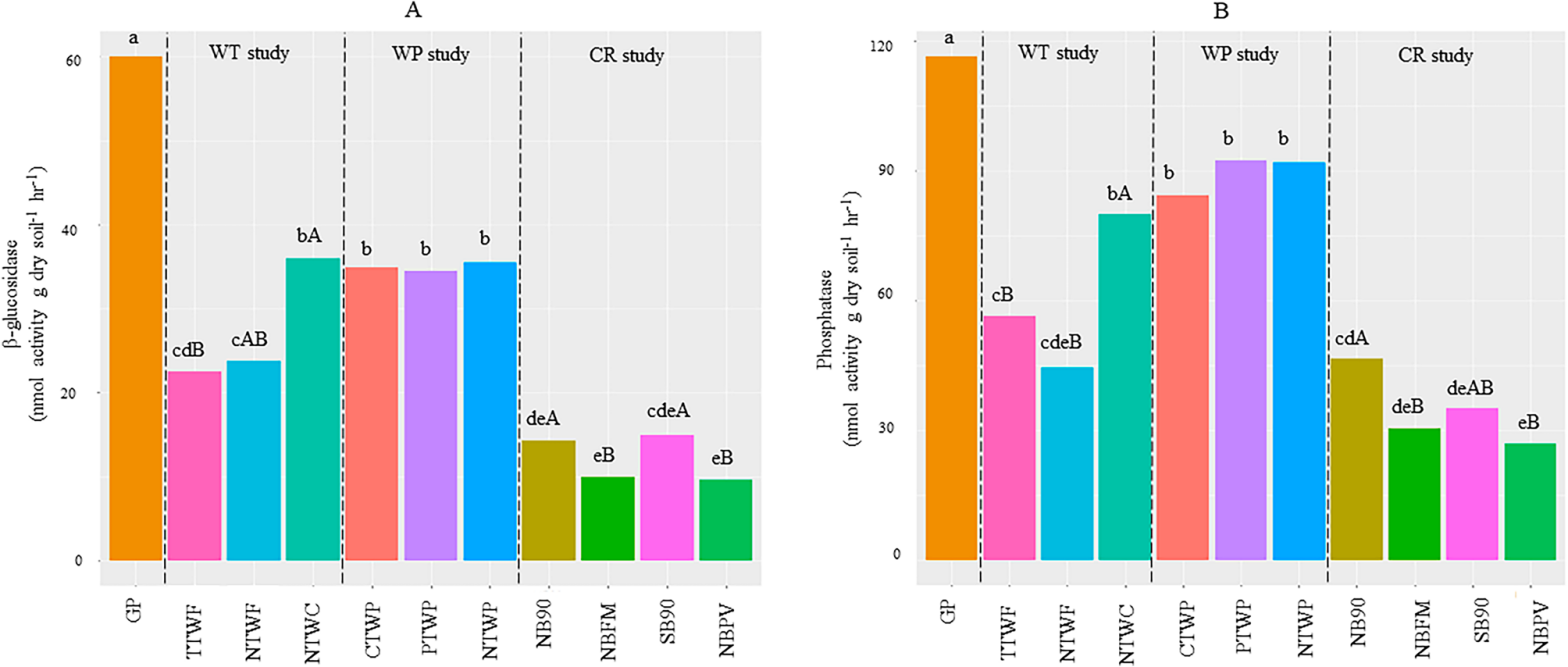
β-glucosidase (**A**) and Phosphatase (**B**) activities in various long-term experiments. Different lowercase letters represent significantly different means across all field studies at *p* ≤ 0.05. Different uppercase letters indicate significantly different means within each study at *p* ≤ 0.05. No uppercase letters mean no statistical significance.

Potential carbon mineralization (1d-CO_2_) showed significant responses to various management practices and varied from 8.8 to 94.1 mg CO_2_–C kg^−1^ (Fig. 1). The highest 1d-CO_2_ was observed in unmanaged GP systems while the lowest were observed in all treatments of CR study. Within CR study, NB90 showed highest 1d-CO_2_. Apart from the GP and treatments of CR study, all other treatments from WT and WP studies showed similar 1d-CO_2_. Across all field studies, 1d-CO_2_ in WT, WP, and CR studies averaged 53%, 51% and 87% lower than GP systems, respectively (Fig. 1). Like 1d-CO_2_, GP showed highest activity for β-glucosidase, and phosphatase (Fig. 2a,b). For β-glucosidase and phosphatase, the lowest activities were observed in all the treatments of the CR study (NBFM, NB90, SB90, and NBPV) (Fig. 2a,b). In WP study, treatments showed no differences in β-glucosidase and phosphatase activities. In WT study, NTWC showed highest β-glucosidase and phosphatase activities. In CR study, NB90 and SB90 showed highest β-glucosidase and phosphatase activities than NBFM and NBPV (Fig. 2b).

### Bacterial community structure

**Figure 3:**
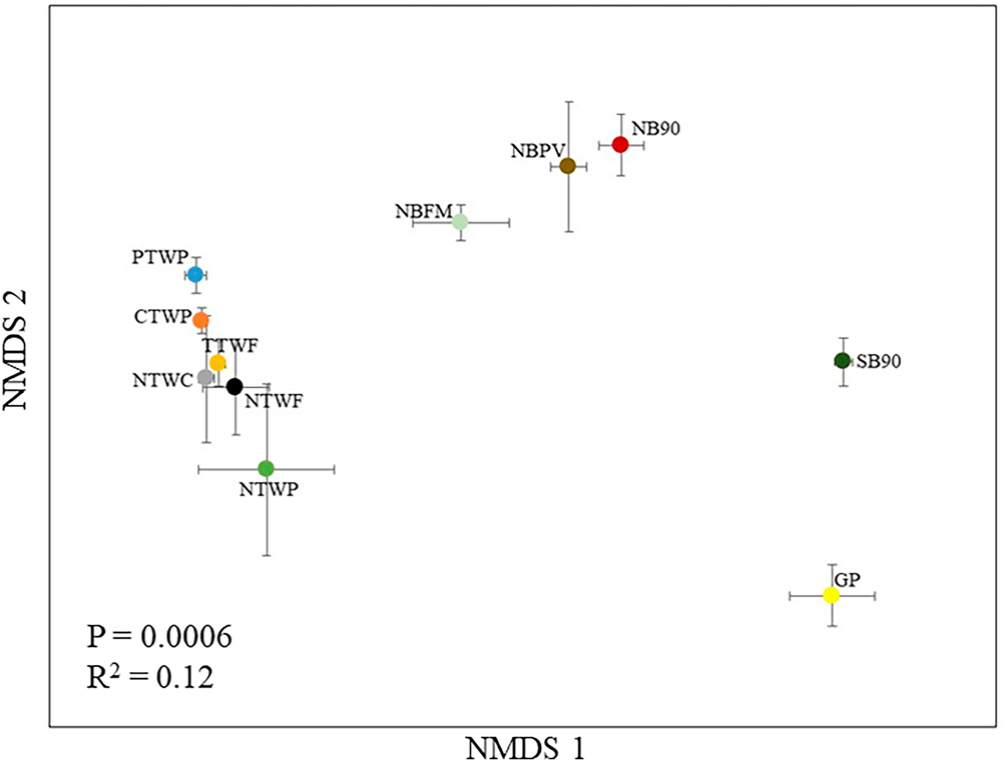
Two dimensional NMDS ordination of bacterial community structure in soils from various long-term experiments. The clustering of samples is based on Bray–Curtis dissimilarity index analyzed using permutational multivariate analysis of variance.

We conducted the bacterial community profiling of soil samples to investigate the effects of various management practices on bacterial community structure. The PERMANOVA results confirmed the significant effect of management strategies on bacterial communities (*p* = 0.0006; R^2^ = 0.12) (Fig. 3). The Non-metric multidimensional scaling ordination plot was used to visualize the structure of dataset using relative distance of the observations and showed that unmanaged GP and burning stubble (SB90) differed from other treatments and from each other. Across all studies, the CR study treatments (NBFM, NBPV, and NB90) also differed from all the other treatments. However, the treatments from WT and WP studies, clustered together and the bacterial community structure looked spatially similar for all these treatments (Fig. 3).

**Figure 4:**
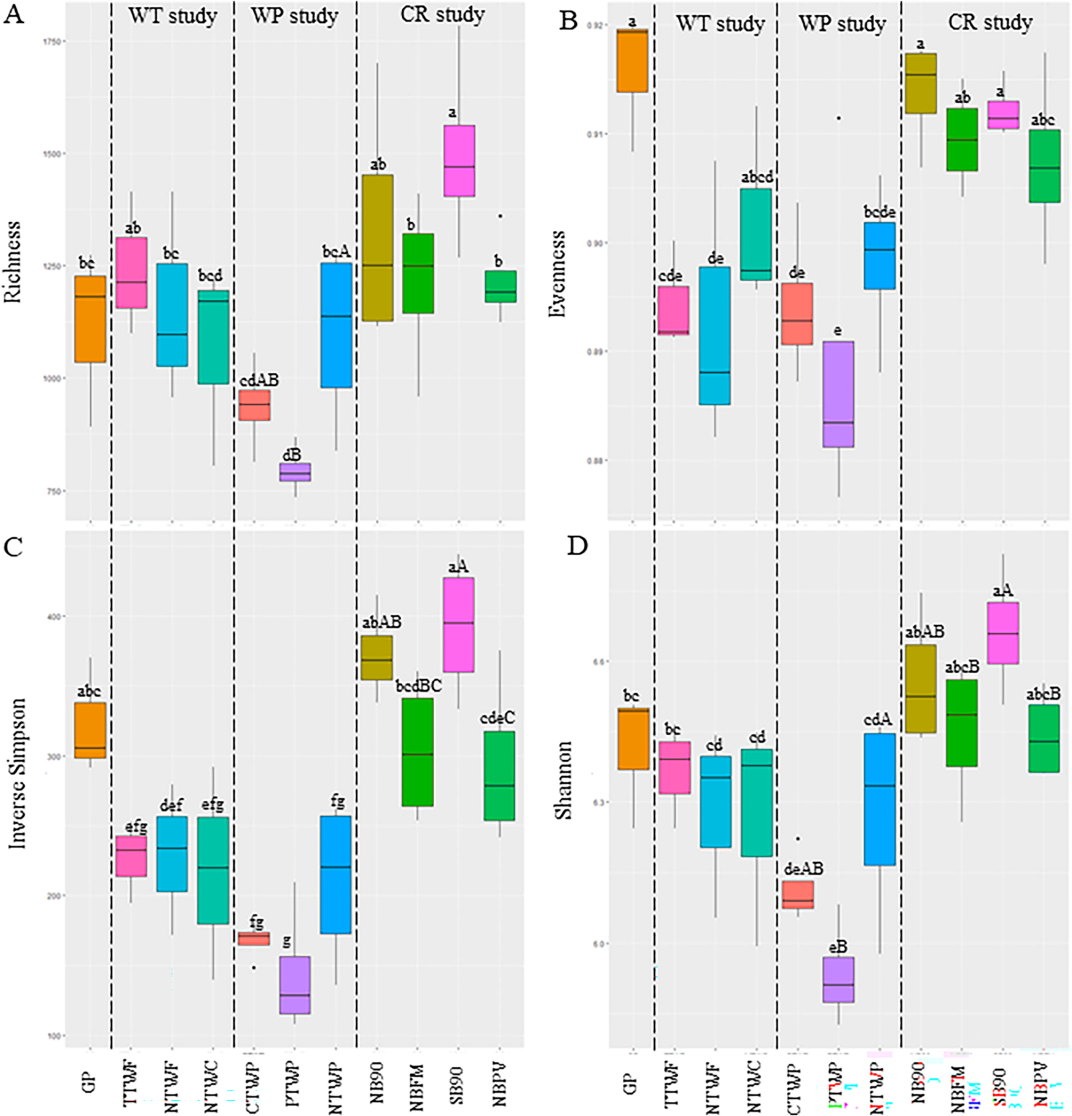
Diversity indices including species richness (**A**), evenness (**B**), inverse Simpson (**C**), and Shannon index (**D**) in various long-term experiments. Different lowercase letters represent significantly different means across all field studies at *p* ≤ 0.05. Different uppercase letters indicate significantly different means within each study at *p* ≤ 0.05. No uppercase letters mean no statistical significance.

Various bacterial diversity indices including richness, evenness, inverse Simpson, and Shannon were also determined and all were significantly influenced (*p* < 0.05) by the management practices (Fig. 4). The management practices significantly influenced the bacterial diversity indices and in general, CR study showed higher diversity than WT and WP studies. Across all studies, SB90 showed the highest and PTWP showed the lowest richness among all the treatments (Fig. 4a). No significant differences among treatments were observed in WT and CR studies, however, in WP study, NTWP showed the highest richness. Similar to richness, inverse Simpson and Shannon indices followed the same trends (Fig. 4c,d) with SB90 showing the highest values. Within each study, the inverse Simpson did not show significant differences among treatments in WT and WP studies but in CR study, SB90 showed highest and NBPV showed the lowest, respectively. Similar responses were also observed in Shannon index. Interestingly, GP system showed the highest evenness along with NTWC, NB90, NBFM, SB90, and NBPV treatments. The lowest evenness was observed in PTWP of the WP study (Fig. 4b).

**Figure 5:**
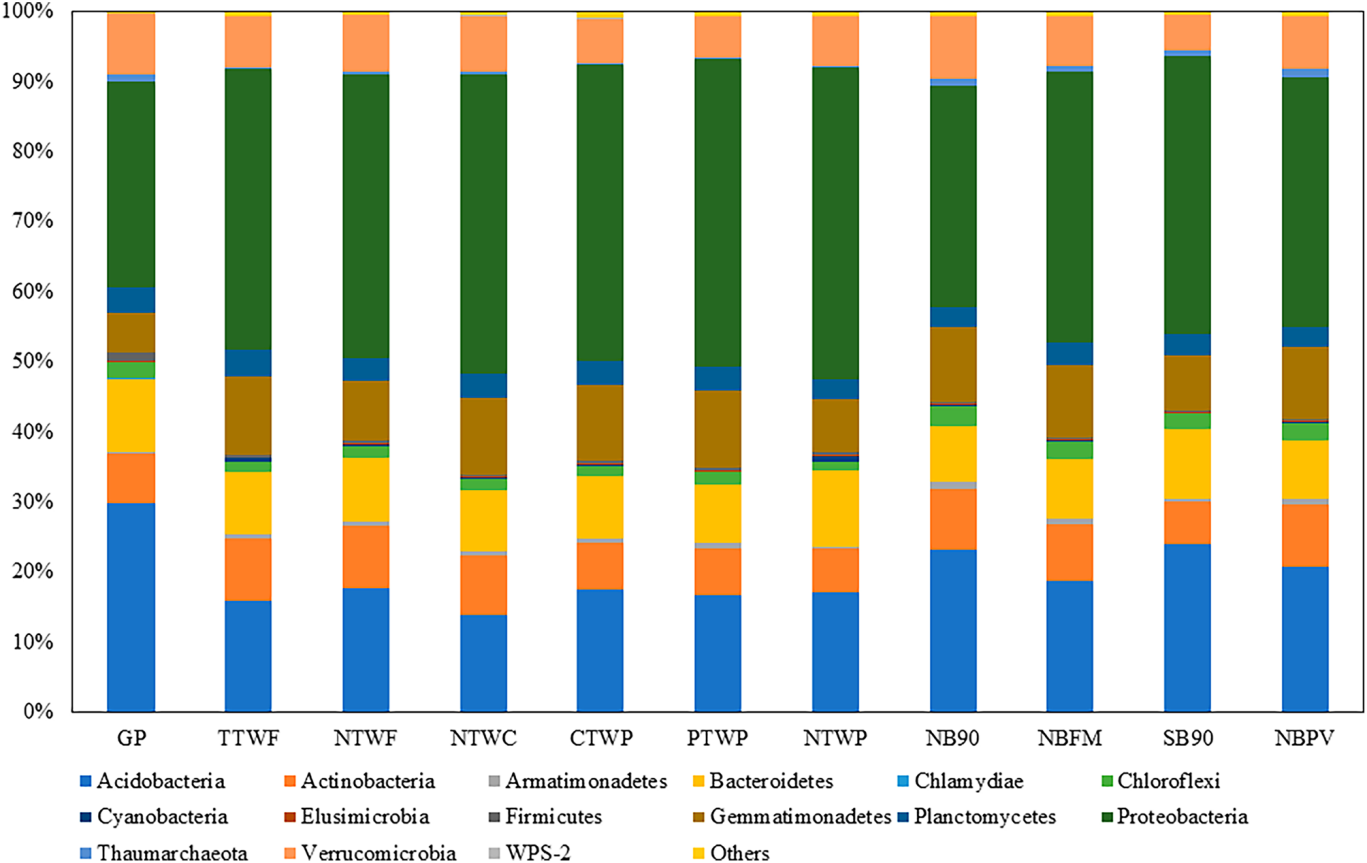
Relative abundance of the dominant phyla in soils from various long-term experiments.

The bacterial community structure at the phylum level showed that Acidobacteria, Actinobacteria, Bacteroidetes, Gemmatimonadetes, Proteobacteria, and Verrucomicrobia comprised the predominant phyla (Fig. 5). The relative abundance of Acidobacteria was significantly higher in GP, NB90, SB90, and NBPV treatments than other treatments (*p* < 0.0001), while Actinobacteria did not differ among the treatments. We observed significant differences in relative abundance of Armatimonadetes with highest in NB90, NBFM, and NBPV while lowest in GP and NTWP treatments. All the treatments from CR study showed the highest relative abundance of Chloroflexi while GP, WT, and WP treatments showed the lowest abundance. The GP treatment exhibited the highest relative abundance of Firmicutes followed by NTWP and the lowest in NTWC. The relative abundance of Gemmatimonadetes was found to be lowest in the GP (*p* = 0.005). No differences were observed in Planctomycetes and Proteobacteria abundances among the treatments. All the treatments from CR study and GP had the highest abundance of Thaumarchaeota while the lowest were found in WT and WP study treatments.

In addition to the phylum level, we looked at the indicator species of each treatment at family level from all the treatments. The most abundant indicator species under GP were Blastocatellaceae, Pyrinomodaceae, Chitinophagaceae, Flavobacteriaceae, Nitrososphaeraceae, Chthoniobacteraceae, Rubrobacteriaceae, and Xanthobacteraceae. In WT study, the most abundant indicators were Gemmatimodaceae, Sphingomodaceae, Bdellovibrioceae, Streptomycetaceae, and Pedosphaeraceae in NTWC and NTWF treatments while in TTWF only Gemmatimodaceae was found. Micromonosporaceae, Fimbriimodaceae, Vermiphilaceae, Caulobacteraceae, and Sphingomodaceae were common in CTWP while in NTWP, the common indicators were Acidobacteriaceae, Solibacteraceae, and Micropepsaceae. The PTWP of WP study showed completely different set of indicator species including Nocardioidaceae, Chitinophagaceae, Bdellovibrioceae, and Xanthomodaceae. The CR study treatments showed different indicators for the treatments. The common indicators for NBFM were Pyrinomodaceae, Pirellulaceae, Gemmatimodaceae, Micropepsaceae, and Xanthobacteraceae while for SB90 were Koribacteraceae, Thermoarobaculaceae, Gaiellaceae, Nocardioidaceae, Rhodomicrobiaceae, and Desulfarculaceae. For NBFM, the common indicators were Pyrinomodaceae, Gemmatimodaceae, Micropepsaceae, and Xanthobacteraceae, while for NBPV, Haliangiaceae was a common indicator.

**Figure 6:**
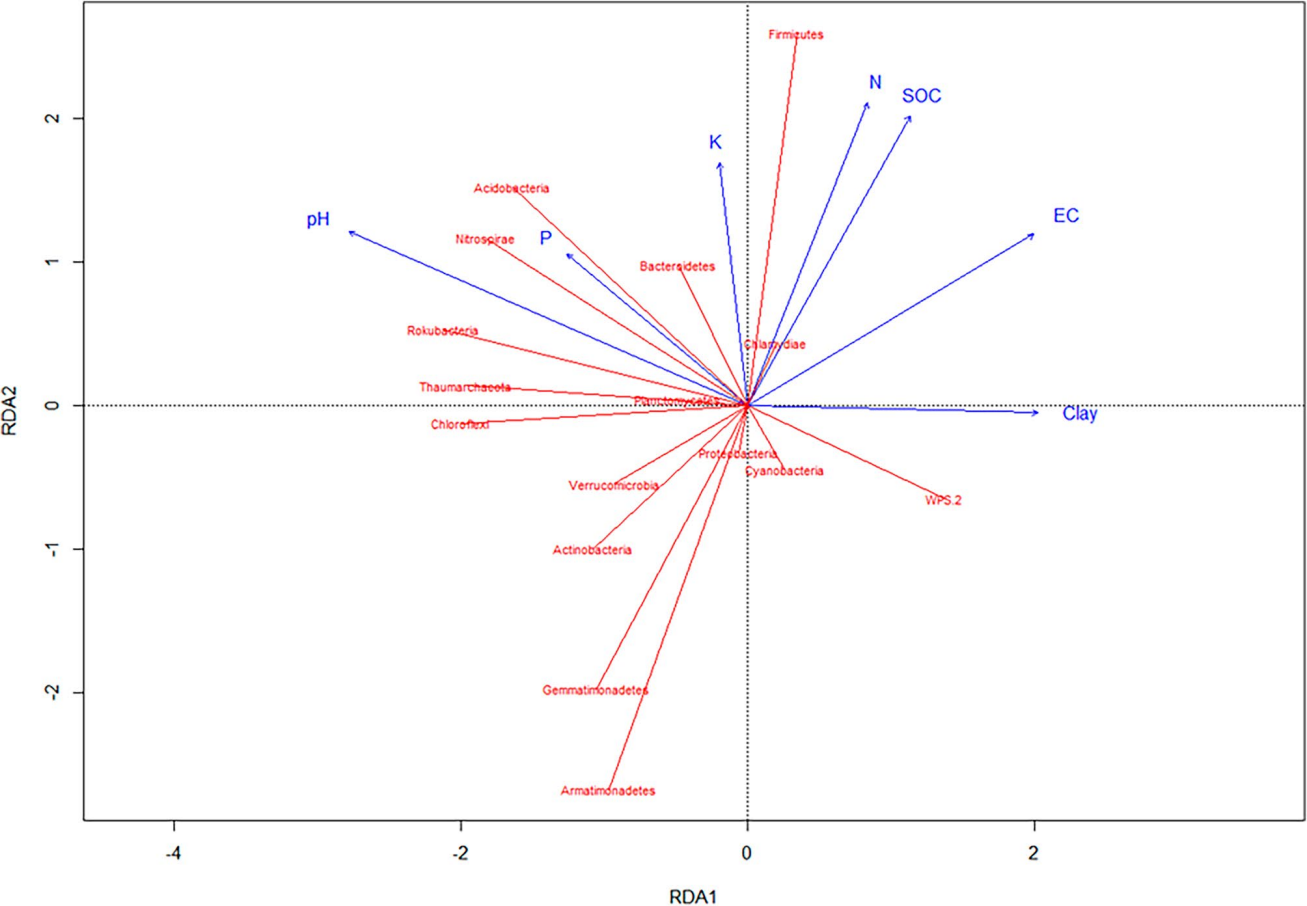
Redundancy analysis plot for the soil parameters and bacterial community from various long-term experiments. The RDAs were implemented using R software.

To explore the relationship between bacterial phyla and soil characteristics, redundancy analysis (RDA) was used. There was an association between available P and K, pH, SOC, and abundance of Acidobacteria, Nitrospirae, Rokubacteria, and Bacteroidetes (Fig. 6). Firmicutes showed a close association with SOC and TN. Clay content showed a negative relationship with most of the bacterial phyla. Pearson correlation between soil properties and bacterial phyla is shown in Table 3. Acidobacteria was positively influenced by available P (r = 0.51), available K (r = 0.39), and pH (r = 0.72). Similarly, available P also had a positive relationship with Bacteroidetes (r = 0.39), Nitrospirae (r = 0.38), Proteobacteria (r = 0.34) and Rokubacteria (r = 0.43) (Table 3). Interestingly, available K had significantly negative impacts on Actinobacteria, Armatimonadetes, Gemmatimonadetes, and Verrucomicrobia. The other phyla significantly positively influenced by soil pH were Chloroflexi (r = 0.62), Nitrospirae (r = 0.71), and Rokubacteria (r = 0.75). Most importantly, we observed that SOC and TN showed similar responses in relationships with bacterial phyla. Actinobacteria (SOC, − 0.49; TN, − 0.39), Armatimonadetes (SOC, − 0.65; TN, − 0.66), and Gemmatimonadetes (SOC, − 0.61; TN, − 0.59) showed significantly negative relationships with SOC and TN while Firmicutes showed a positive relationship (SOC, 0.61; TN, 0.67) (Table 3).

**Table 3:** Pearson correlation between bacterial community and soil properties.

	Phosphorus	Potassium	pH	EC	Clay content	SOC	TN
Acidobacteria	**0.51**^†^	**0.39**	**0.72**	− 0.07	− **0.43**	0.09	0.19
Actinobacteria	− 0.02	− **0.35**	0.09	− 0.15	− **0.54**	− **0.49**	− **0.39**
Armatimonadetes	− 0.24	− **0.48**	− 0.01	− **0.74**	− 0.06	− **0.65**	− **0.66**
Bacteroidetes	**0.39**	0.31	0.21	0.28	− **0.33**	0.13	0.17
Chlamydiae	0.31	0.31	− 0.05	**0.32**	− 0.13	0.18	0.18
Chloroflexi	0.27	0.02	**0.62**	− **0.45**	− **0.44**	− 0.29	− 0.22
Cyanobacteria	− 0.15	− 0.14	− 0.28	0.08	− 0.16	0.01	0.01
Firmicutes	0.04	0.27	0.16	**0.41**	− 0.08	**0.61**	**0.67**
Gemmatimonadetes	− 0.04	− **0.35**	0.02	− **0.38**	− **0.32**	− **0.61**	− **0.59**
Nitrospirae	**0.38**	0.25	**0.71**	− 0.27	− **0.39**	0.03	0.09
Planctomycetes	0.27	0.01	0.07	0.18	− **0.52**	− 0.13	− 0.01
Proteobacteria	**0.34**	0.16	− 0.06	0.25	− 0.17	− 0.12	− 0.13
Rokubacteria	**0.43**	0.22	**0.75**	− **0.41**	− **0.38**	− 0.15	− 0.11
Verrucomicrobia	− 0.13	− **0.35**	0.09	− 0.24	− **0.49**	− 0.27	− 0.17

^†^Bold numbers indicate significant correlation with *p* ≤ 0.05.

## Discussion

### Response of soil properties to management practices

In this study, we investigated the effects of a suite of long-term management practices on soil chemical and biological properties. Our finding of higher SOC and TN under unmanaged GP systems (Table 2) could be due to the high amounts of dense fibrous grass root turnover which have higher lignin and hemicellulose contents^44, 45^ and these grasses are perennial and undisturbed which may lead to a more continuous supply of root exudates. Among agricultural systems, generally NT systems (e.g., NTWP) having higher SOC content than conventional tillage treatments (Table 2) are also reported in previous studies^46–48^. Typically, tillage practices such as moldboard plowing that severely disturb and invert the soil, accumulate less SOC than chisel till^46, 49, 50^ as observed in our results. Interestingly, we observed higher SOC in treatments involving SB90 (spring residue burning with 90 kg N ha^−1^) application than in other treatments from the CR study. This may have two plausible reasons: (i) deposition of ash in soils^51^, and (ii) maybe lower degradability of the C in burnt residues. Dutta, et al.^52^ also reported that burnt residue decomposition over years leads to higher SOC in the rice–wheat rotation system they assessed. In addition, burning residue in spring season protects the soil aggregates during the prior winter months from the precipitation because most to all precipitation in the region occurs in the winter months^53^ potentially physically protecting SOC. Similar explanation could also be valid for higher TN in SB90 treatment^48^. Likewise, higher available P and K in SB90 can be attributed to higher nutrient retention due to higher SOC. Contrary to our expectations, the WT study, that was established almost 16 years ago from the time of sampling, did not show significant differences among treatments in SOC, TN, P, K, pH, and EC (Table 2), suggesting that these practices (chisel till, no-till and no-till with cover crops) may take longer than that to equilibrate and show treatment effects, especially under the lower biomass producing dryland wheat-fallow systems. It is also important to note that these plots are in the low precipitation region of eastern Oregon which also has low primary productivity than the other two experimental sites. In addition, some of the previous studies showed increased SOC and TN in the WT study under NT systems (e.g., NTWC and NTWF) when sampled within the top 0–10 cm profile^29^. However, the current study was conducted using samples from 0 to 15 cm profile^54^ which could potentially dilute the treatment effects.

### Responses of carbon mineralization potential to management practices

In accordance with our expectations, unmanaged GP systems had higher carbon mineralization potential (1d-CO_2_) (Fig. 1) and similar trends were observed in enzyme activities of β-glucosidase and phosphatase enzymes (Fig. 2). β-glucosidase participates in the decomposition of polysaccharides such as cellulose and hemicellulose while phosphatase enzyme helps in breaking down organic P in soil organic matter and releasing available P. Both enzyme activity tests showed similar trends and were highest in the unmanaged GP systems. Generally, enzyme activity is directly related to SOC content which supports our results as GP systems had the highest SOC^55, 56^. For the WT study, no tillage treatments (NTWC and NTWF) had higher enzyme activity than chisel till (CTWP). It is widely reported that β-glucosidase activity increases when soil disturbance is reduced and SOC is increased^57, 58^. The NTWC showed the highest β-glucosidase enzyme activity and that could mainly be due to the higher biomass production and decomposition of legume cover crop residues. In the WP study, tillage intensities did not differentiate in β-glucosidase activity (Fig. 2a) which could be due to the presence of leguminous pea crop which provided substrate supply of root exudates and biomass C and N inputs for the enzymes to act on^59, 60^. For the CR study, NB90 and SB90 showed the highest β-glucosidase activity (Fig. 2a) which could probably be due to higher SOC content and C addition from higher biomass due to higher N application in these treatments. In the NB90, the residue is left as such that adds the biomass and in SB90, wheat biomass stayed in the field for a significant amount of time before burning^48^. Phosphatase activity was mostly related to available P levels in soils across different treatments and similar phosphatase activities were also reported by^61–63^. In the WT study, undisturbed soils with cover crops had elevated phosphatase activity due to higher substrate availability leading to elevated metabolism of soil microorganisms^20, 64^. Trends were similar between β-glucosidase and phosphatase in the CR study (Fig. 2b).

### Response of soil bacterial community structure

Bacterial diversity is critical for the function, integrity, and sustainability of soils^65^. It is important to understand how bacterial communities harbored in soil are affected by agricultural management practices and how key players can be targeted for sustainable ecological intensification. Higher bacterial diversity confers ecosystem resilience in the face of any kind of disturbance. Resilience is defined as the rate at which a microbial community returns to its original composition after being disturbed^66^. In this study, bacterial diversity indices including richness, evenness, Shannon index, and inverse Simpson were calculated. Interestingly, GP which exhibited the highest SOC, showed lower richness than NB90 and SB90, however, higher than more soil-intensive treatments like CTWP and PTWP (Fig. 4a). This could be explained by the fact that higher nutrient availability in GP drives a relatively less metabolically diverse r-selected microbes^67^. Perhaps this is the reason why NTWP treatment showed the highest richness than other treatments in the WP study. Evenness followed the same pattern as richness except that here unmanaged GP also showed the highest species evenness (Fig. 4b) which indicates that no or low degree of disturbance leads to more species evenness. Our data suggest that high SOC and TN in GP could be a driver of higher species evenness^68–70^. Inverse Simpson index that determines the changes in highly abundant species and Shannon index which represents changes in rare species were highest in SB90, NB90, and GP, however, similar to the other two indices, this was found to be lowest in treatments from the WP study (Fig. 4c,d). The difference between the treatments was not conclusive within experiments. It is interesting to see that GP system exhibits a better functioning and healthier soil but the diversity indices except evenness were not the highest in GP. This indicates functional redundancy of microbes in these systems meaning even though the microbial diversity is lower in these systems than other treatments, the diversity loss is compensated by other microbes capable of similar functioning^71^. Our results indicate that perhaps species evenness is a better indicator of soil health than other diversity indices and that increased diversity does not always contribute to microbial functionality.

Unmanaged GP exhibited a higher abundance of Acidobacteria and Firmicutes (Fig. 5) which are characterized as k-strategists and are responsible for the breakdown of C-rich compounds in soil such as cellulose, hemicellulose, and lignin^72^. Previous studies reported that Firmicutes produce β-glucosidase enzyme to break down plant biomass^73, 74^ and similar observation of the highest β-glucosidase activity in GP was evident in our study. Other treatments in which Acidobacteria were high are NB90 and SB90 directly indicating their presence in higher levels of SOC. Contrary to that, the relative abundance of Armatimonadetes and Chloroflexi were lowest in GP, WT, and WP while highest in CR study treatments (Fig. 5). This could be because both these phyla are oligotrophic and are more common in soils with low C content^18, 75, 76^, as seen in our study that lowest SOC was observed in these treatments. This also indicates that Chloroflexi are less competitive in more diverse communities and tend to survive in stressed or nutrient-limited conditions. The relative abundance of Gemmatimonadetes was the lowest in GP systems and typically, this phylum shows an adaptation to low soil moisture conditions or gets outcompeted when soil moisture increases^77^. Here, the highest SOC and no disturbance in GP systems would have been able to retain more soil moisture making it less conducive for the growth of Gemmatimonadetes.

We looked at the indicator species (family level) in each treatment and found that in GP, we found both gram-positive and gram-negative bacteria. As the minimally- managed and grassland systems tend to harbor diverse microbes (as seen in diversity indices), the presence of gram-negative indicators could be due to very high SOC that provided unrestricted substrates availability for microbes. However, the presence of gram-positive indicators that are thick-walled, spore-forming, and oligotrophic indicates that the system is more resilient to disturbances. In the WT study, TTWF showed a gram-negative indicator while NTWF and NTWC showed more diverse set of indicator groups. More specifically, NTWC and NTWF increased the number of indicator families that are oligotrophic. No-tillage leads to soil aggregation^78^ and stable aggregates provide a niche for microbial communities supporting oligotrophic microbes^79^. In the WP study, CTWP treatment fostered only gram-negative copiotrophs while NTWP harbored a diverse set of both gram-positive and gram-negative indicator families. Typically, copiotrophs thrive in systems which have sudden and short-lived pulses of C inputs and CTWP represents such boom-and-bust cycles of C inputs. More destructive tillage treatment PTWP, exhibited indicators which were copiotrophic, anaerobes, and phytopathogens. Burning the straw in the CR study led to the presence of certain thermophilic bacterial families and perhaps they are the ones which contributed to increased diversity in SB90 treatment. Burning could have increased soil temperatures leading to presence of gram-positive and thermophilic bacterial families as well. Similarly, NBFM also supported a mix of r and k strategists, but we found more r strategists in this treatment. It is important to note that this is a wheat-fallow system in which the fallow period could probably be detrimental to bacterial communities^80^ due to no C inputs from plants. It is evident from the Pearson’s correlation table (Table 3) that not only SOC, but also changes in other soil characteristics affect the relative abundance of the bacterial community in different agricultural management systems. There were several significant relationships between bacterial phyla and soil nutrients. Soil clay content was found to be the major controller of phyla abundance followed by pH and other nutrients (Table 3). The changes in Acidobacteria abundance were due to changes in soil pH and available P and K. Similar results were reported by^81–84^. Actinobacteria abundance was observed to be higher in systems with lower SOC and TN (more disturbed systems) indicating their adaptation to disturbed systems or perhaps higher turnover in undisturbed systems. Our results are in concordance with^85, 86^. Bacterial phyla positively influenced by available P were Bacteroidetes, Nitrospirae, Proteobacteria, and Rokubacteria. All of these phyla are gram-negative which are thin-walled and are increasing in abundance with an increase in available P (coming from the fertilizer inputs). Interestingly, most of the phyla showing a positive relationship with available P, showed a negative correlation (both significant and non-significant) with SOC and TN indicating that less soil disturbing practices tend to harbor bacterial taxa that are oligotrophic and can survive future stress. Our results followed a consistent trend in which we found that less disturbing management practices exhibited more diverse bacterial community. This is because less or no disturbance in soils could potentially provide a suitable environment (better moisture and nutrient retention) and pore architecture which promotes habitat to foster microbial communities and enhance overall soil health^84, 87, 88^.

## Conclusion

Unmanaged grasslands, no tillage, and cover crops increased SOC, TN, and available nutrients. Soil health indicators such as carbon mineralization potential and enzyme activities were significantly higher in unmanaged GP systems as compared to wheat systems. Diversity indices were found to be the highest in CR study and GP as compared to WT and WP studies. Bacterial taxa were also influenced by the soil chemical characteristics. The results indicated that conservation practices like no-tillage, cover crops, and unmanaged GP had a higher abundance of oligotrophs and higher evenness than practices that caused more soil disturbance. The data suggests that since conservation practices harbor more diverse and oligotrophic bacteria, these systems are more resilient to stress or disturbance. Further, it would be interesting to conduct in-depth transcriptomic studies to understand the link to microbial activities in these systems while considering both spatial and temporal heterogeneities. Understanding the adaptation and survival strategies of soil bacteria under different management practices may provide useful information to the growers and stakeholders in the adoption of sustainable and conservation practices.

## Data Availability

The datasets generated and/or analyzed during the current study are available in the figshare repository with https://doi.org/10.6084/m9.figshare.20737018 and the sequences analyzed in this manuscript are submitted to National Center for Biotechnology Information Sequence Read Archive with accession number PRJNA762046.
